# Construction, Validation, and Visualization of Two Web-Based Nomograms for Predicting Overall Survival and Cancer-Specific Survival in Elderly Patients with Primary Osseous Spinal Neoplasms

**DOI:** 10.1155/2022/7987967

**Published:** 2022-04-04

**Authors:** Yuexin Tong, Yuekai Cui, Liming Jiang, Yuan Zeng, Dongxu Zhao

**Affiliations:** ^1^The China-Japan Union Hospital of Jilin University, Changchun, Jilin 130000, China; ^2^The First Affiliated Hospital of Wenzhou Medical University, WenzhouZhejiang 325000, China

## Abstract

**Background:**

Primary osseous spinal neoplasms (POSNs) are the rarest tumor type in the spine. Very few studies have presented data on elderly patients with POSNs specifically. The present study was aimed at exploring the prognostic factors and developing two web-based nomograms to predict overall survival (OS) and cancer-specific survival (CSS) for this population.

**Method:**

The data of elderly patients with POSNs was extracted from the Surveillance, Epidemiology, and End Results (SEER) database between 2004 and 2015. Cox regression analyses were performed to determine independent prognostic factors for OS and CSS, these prognostic factors were incorporated to establish nomograms. The discrimination of the nomograms was evaluated by the receiver operating characteristic (ROC) curve and the value of area under the curve (AUC). Calibration curve was plotted to assess the predictive accuracy of model. Decision curve analysis (DCA) was conducted to determine the net clinical benefit. Furthermore, two web-based survival rate calculators were developed.

**Result:**

A total of 430 patients were finally selected into this study and were randomly assigned to the training set (302 cases) and validation set (128 cases). Of these, 289 patients were further considered for the analysis of CSS and were randomized into training set (205 cases) and validation set (84 cases). Based on the results of univariate and multivariate Cox analyses, variables that significantly correlated with survival outcomes were used to establish nomograms for OS and CSS prediction. Two established nomograms demonstrated good predictive performance. In the training set, the AUCs of the nomogram for predicting 12-, 24-, and 36-month OS were 0.849, 0.903, and 0.889, respectively, and those for predicting 12-, 24-, and 36-month CSS were 0.890, 0.880, and 0.881, respectively. Two web-based survival rate calculators were developed to estimate OS (https://research1.shinyapps.io/DynNomappOS/) and CSS (https://research1.shinyapps.io/DynNomappCSS/).

**Conclusion:**

Novel nomograms based on identified clinicopathological factors were developed and can be used as a tool for clinicians to predict OS and CSS in elderly patients with POSNs. These models could help facilitate a personalized survival evaluation for this population.

## 1. Introduction

Primary osseous spinal neoplasms (POSNs) are relatively rare conditions comprising only 10% or less of all bone neoplasms [1]. Common histologic entities of POSNs include chordomas, osteosarcomas, chondrosarcomas, and Ewing sarcomas [2–4]. Clinical signs and symptoms of these tumors are diverse and lack specificity. Local pain is the most common symptoms and is presented in up to 85% of patients with POSNs, other symptoms include radicular pain, spinal instability, and pathological fracture [5, 6]. Due to these, manifestations can be easily confused with degenerative spinal disease and the rarity of POSNs, the diagnosis of these patients is frequently delayed, thereby resulting in low quality of life and poor prognosis.

With the aging of the global population, human life expectancy has been continuously increasing over the past decades, by 2050, over 2 billion (22%) of the world's population will be comprised of people over 60 years of age and, of these, 402 million individuals will be over 80 years, the effective management and precision medicine of elderly cancer patients will be a major challenge for the coming years [7]. Elderly patients usually represent a heterogeneous population as they not only tend to suffer from various comorbidities but are also characterized by decline in physiological compensatory capacity, varying degrees of functional disability, and a reduced ability to resist side effects of therapy [8–10]. A previous study suggested that elderly patients had shorter survival than their younger counterparts [11]. This did also echo why elderly patients act as an important component of the overall population diagnosed as POSNs therefore needs to be given more attention. In a latest research, Helenius and Krieg have studied the early diagnosis, treatment principles, and prognostic factors regarding primary malignant bone tumors of the spine and pelvis in children [12], while a gap of knowledge still remained with respect of prognostic assessment for elderly patient with POSNs.

Therefore, this study was aimed at developing predictive nomograms and web-based survival rate calculators that can dynamically predict the long-term overall survival (OS) and cancer-specific survival (CSS) of elderly patients with POSNs based on a large population derived from the Surveillance, Epidemiology, and End Results (SEER) database.

## 2. Materials and Methods

### 2.1. Data Source and Study Population

**Figure 1:**
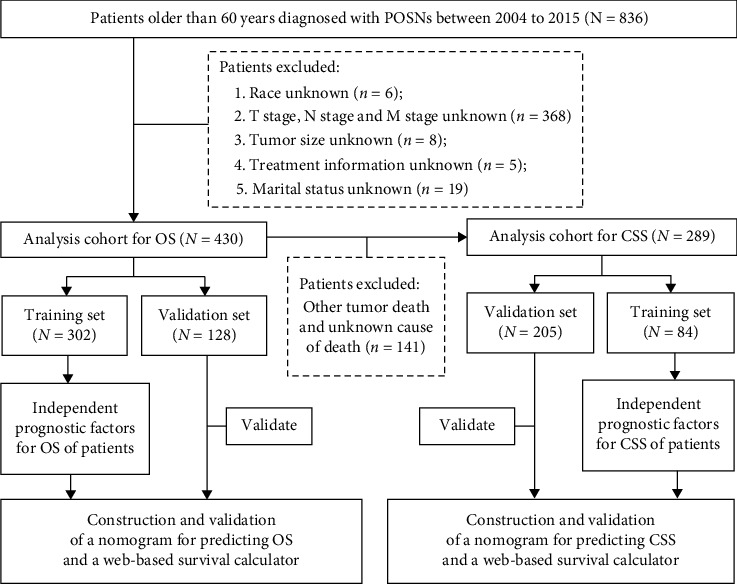
The flowchart of selection procedure of elderly patients with POSNs and study design.

The comprehensive data of elderly patients with POSNs from 2004 to 2015 were retrieved from the SEER database (http://seer.cancer.gov/) using the SEER∗Stat software version 8.3.6 (https://seer.cancer.gov/seerstat/software/). Due to the unidentified data in the SEER database, this analysis is exempt from medical ethics review and does not require informed consent. The inclusion criteria were as follows: (1) patients were histologically diagnosed as primary malignant bone tumor between 2004 and 2015; (2) site limited to the osseous spine (vertebral column; sacrum/pelvis); (3) patients were older than 60 years old; and (4) patients with complete follow-up. The patients who were diagnosed by death certificate or autopsy and with incomplete information including race, TNM stage, tumor size, treatment information, and marital status were excluded. The flow chart for patient selection and research process is shown in Figure 1. The following demographic and clinicopathological variables were included in our analysis: age, sex, race, histological type, grade, T stage, N stage, M stage, marital status, tumor size, surgery, radiotherapy, chemotherapy, survival months, vital status, and cause of death. The X-tile program (version 3.6.1) provided the optimal cutoff point of age and tumor size and then transformed continuous variables into categorical variables [13]. The primary outcomes in our study were designed as OS and CSS. OS was defined as the time from diagnosis to death regardless of any cause, and CSS was defined as the time from diagnosis to death from POSNs.

## 3. Statistical Analysis

Data analysis was performed with the SPSS software (SPSS for Windows 26.0, SPSS, Inc., Chicago, IL) and R software (version 4.1.1). The selected patients were divided randomly into training set (70%) and validation set (30%). A chi-squared test was used to determine between-group differences in baseline characteristics. All variables were firstly analyzed by univariate Cox analysis, those with *P* < 0.05 will be included in the multivariate Cox analysis to obtain statistically significant variables, which were determined as independent prognostic factors for OS and CSS in elderly patients with POSNs. Afterward, the prognostic nomograms for OS and CSS were created separately based on these identified predictors by the “rms” package in R software. Meanwhile, the receiver operating characteristic (ROC) curves with the value of area under the curve (AUC) and calibration curves were generated to evaluate the accuracy of the nomogram. Clinical usefulness was evaluated by decision curve analysis (DCA). Finally, according to the optimal cutoff value of total points determined by the X-tile software, all patients were divided into high-, medium-, and low-risk subgroup. And the Kaplan-Meier survival analysis with a log-rank test was conducted to compare the survival difference between subgroups. Finally, two web-based survival rate calculators were further established based on the nomograms using the “Dynnom” package.

## 4. Results

### 4.1. Patient Characteristics

According to the inclusion and exclusion criteria, a total of 430 patients were finally selected for our study and included in the analysis of OS, they were randomized into training set (*n* = 302) and validation set (*n* = 128). Of these, 289 patients were further considered for the analysis of CSS and randomized into training set (*n* = 205) and validation set (*n* = 84). The baseline clinicopathological characteristics of patients in the OS group are shown in Table 1. Among elderly patients with POSNs, a higher proportion of patients were male (57.44%) than female (42.56%). In relation to race distribution, most patients were white people (87.21%). As for the histological type, chordoma accounted for 42.79%, accounting for the highest, followed by chondrosarcoma (32.09%), osteosarcoma accounted for the lowest, 11.63%. In terms of tumor characteristics, 206 cases (47.91%) presented with the tumor size of 6-12 cm, 227 cases were in stages T1 (52.79%), 415 cases were in stage N0 (96.51%), and 383 cases were in stage M0 (89.07%). Most of these patients were married (65.12%). Additionally, over half of the elderly patients with POSNs received surgery (62.56%), conversely, most of the patients did not undergo chemotherapy (84.88%) or radiotherapy (63.95%). The baseline clinical pathological characteristics of patients in the CSS group are shown in Table 2.

### 4.2. Independent Prognostic Factors for OS and CSS

**Figure 2:**
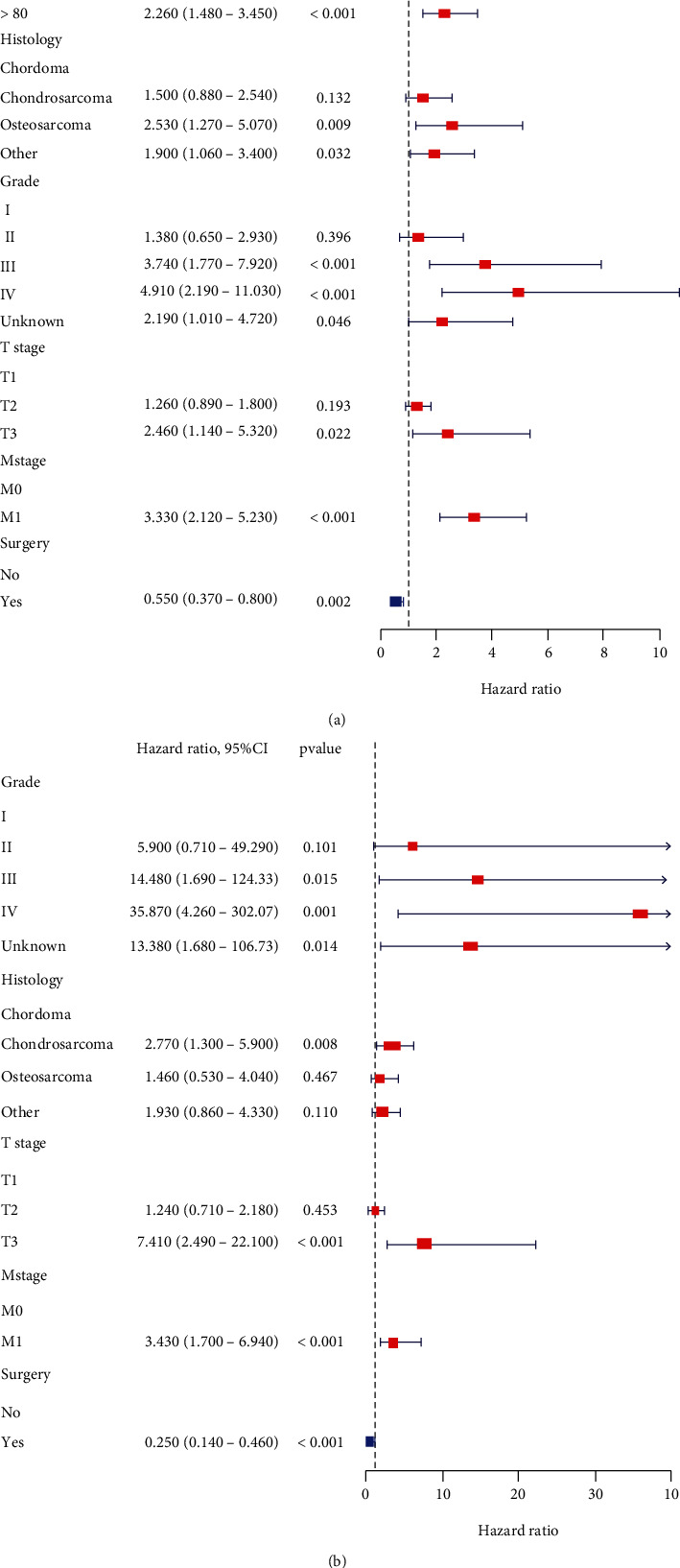
Forest plots depicting the effects of different prognostic factors for OS (a) and CSS (b).

Univariate and multivariate Cox proportional hazard regression analyses were performed to determine the independent prognostic factors for OS and CSS of elderly patients with POSNs. The results of the univariate Cox analysis are shown in Table 3. The forest plots showing the results of the multivariate Cox analysis are shown in Figure 2. Age, grade, histological type, T stage, M stage, and surgery were identified as independent prognostic factors of OS; and grade, histological type, T stage, M stage, and surgery were identified as independent prognostic factors of CSS.

### 4.3. Construction and Validation of Nomograms

**Figure 3:**
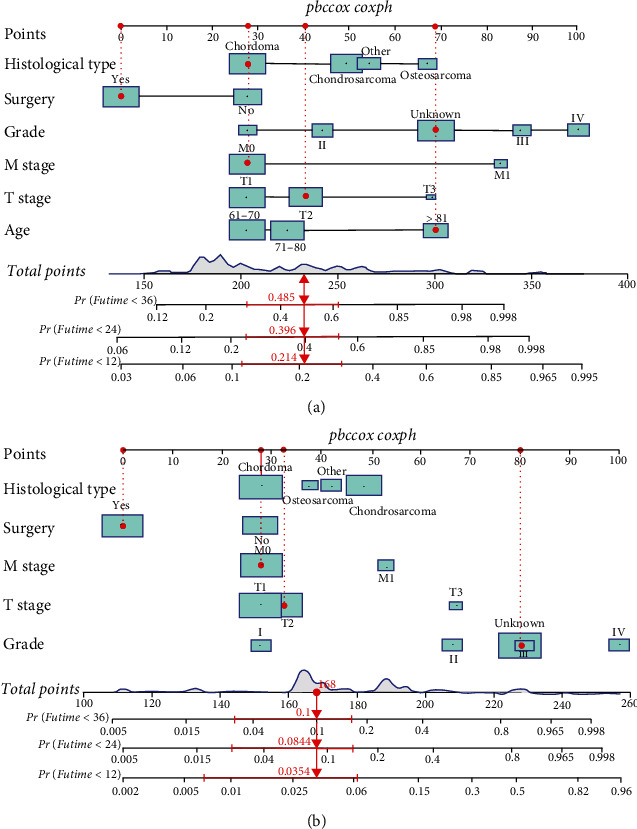
Nomograms predicting 12-, 24-, and 36-month OS (a) and CSS (b) of elderly patients with POSNs.

Based on the independent prognostic factors for OS and CSS derived and identified from the Cox proportional hazard regression analyses, we built and interpreted two nomograms (Figure 3) for predicting the survival rate. An individual patient' s value is located on each variable axis, and a line is drawn upward to determine the number of points received for each variable value. The sum of these numbers is located on the total point axis, and a line is drawn downward to the survival axis to determine the probabilities of OS and CSS at 12-, 24-, and 36 months, respectively. In our new visualized nomogram, the blue boxes below the name and the grey color block on the total point axis represent the sample size, which shows the demographic statistics of the elderly patients with POSNs and the population distribution of the prognosis. For example, a patient is more than 81 years of age and this patient is characterized as a stage T2 chordoma without distant metastatic disease at the time of presentation and has undergone surgery. Consequently, the total points of OS are 233 and the probability OS at 12-, 24-, and 36-month is 0.786, 0.604, and 0.515, respectively.

**Figure 4:**
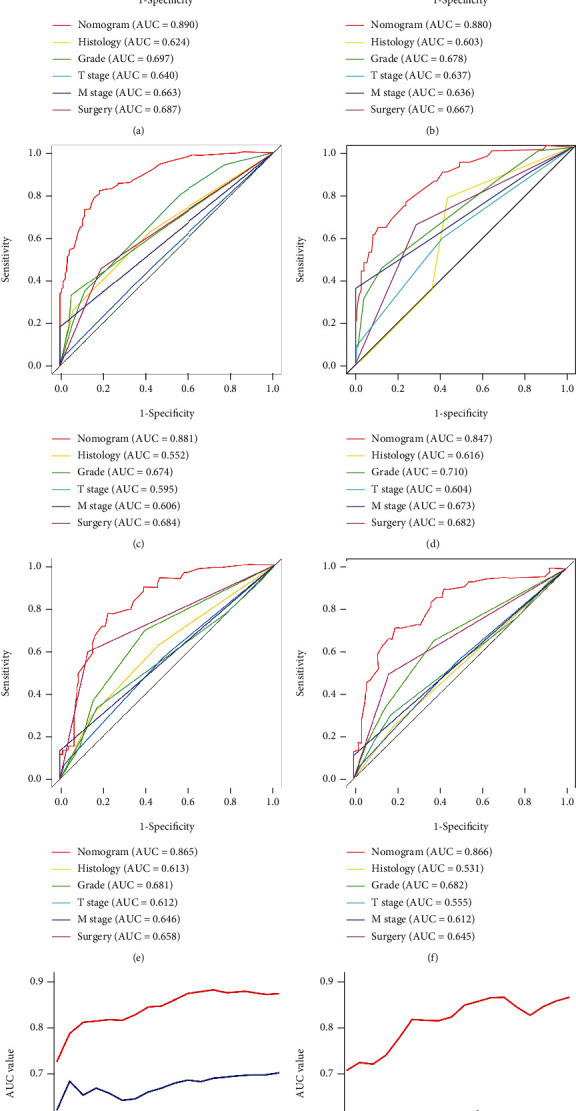
ROC curves of the nomogram for 12-month (a), 24-month (b), and 36-month (c) OS prediction in the training set and for 12-month (d), 24-month (e) and 36-month (f) OS prediction in the validation set. The time-dependent ROC curves of the nomogram for OS prediction in the training set (g) and validation set (h).

**Figure 5:**
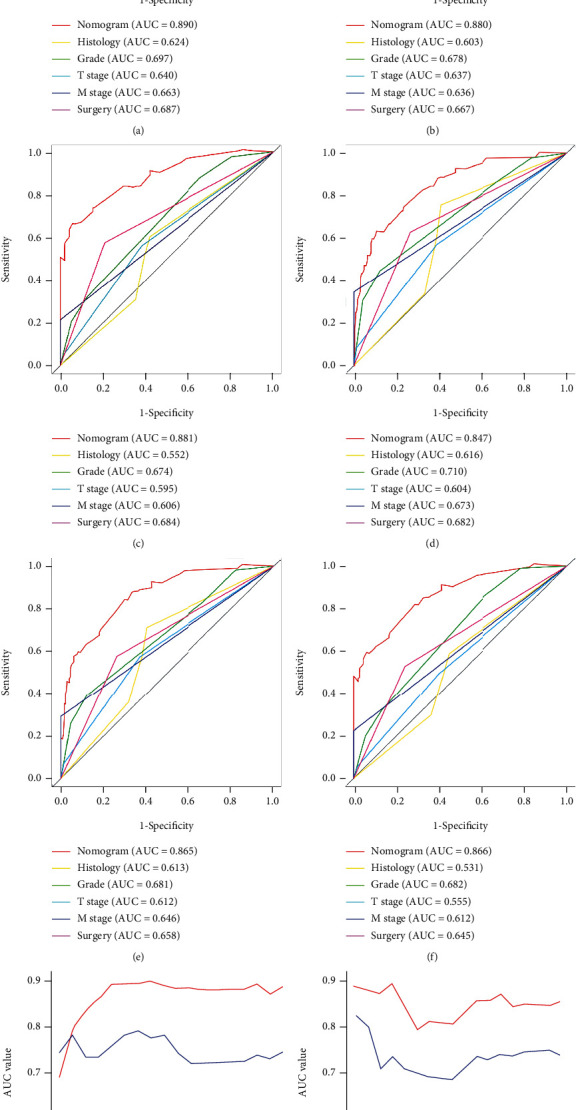
ROC curves of the nomogram for 12-month (a), 24-month (b), and 36-month (c) CSS prediction in the training set and for 12-month (d), 24-month (e), and 36-month (f) CSS in the validation set. The time-dependent ROC curves of the nomogram for CSS in the training set (g) and validation set (h).

**Figure 6:**
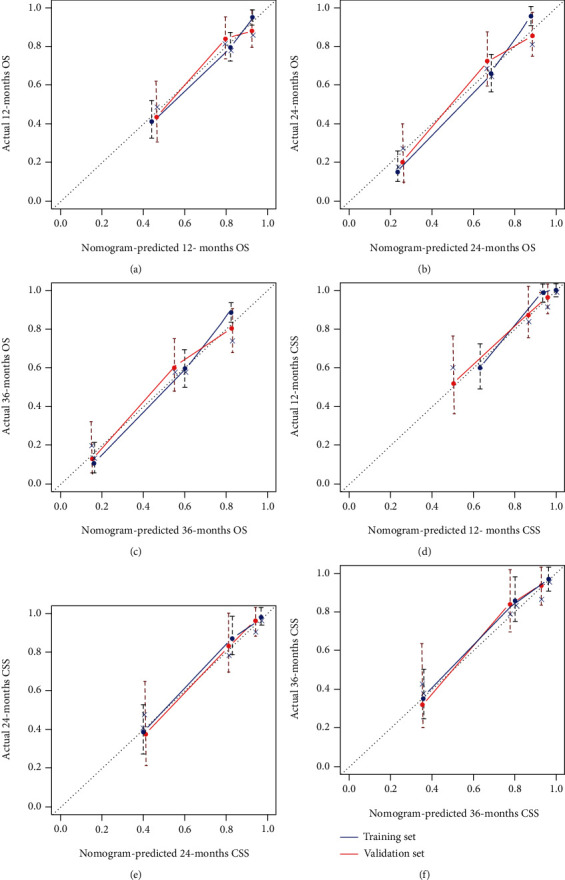
Calibration curves of the nomograms for 12-month (a), 24-month (b), and 36-month (c) OS prediction and for 12-month (d), 24-month (e), and 36-month (f) CSS prediction.

**Figure 7:**
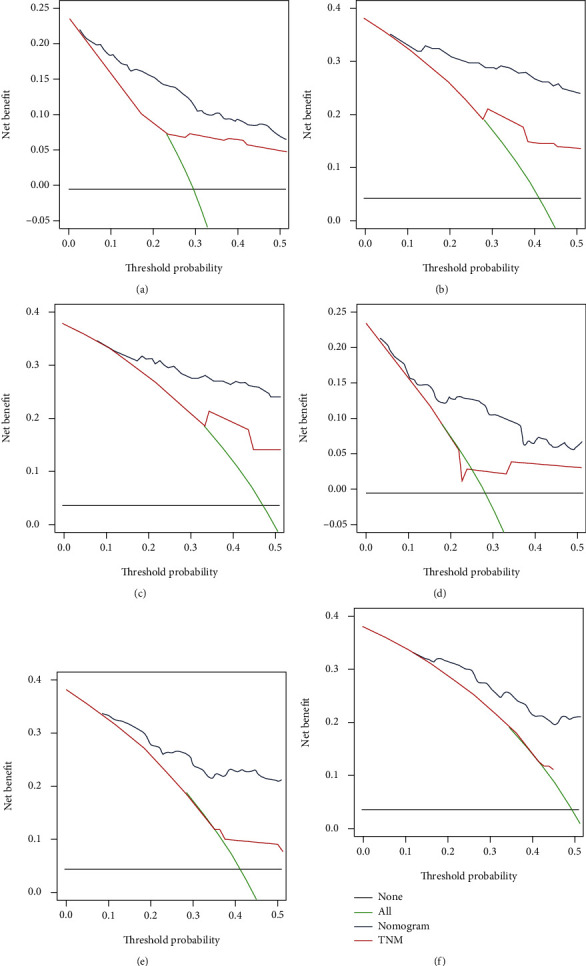
DCA of the nomograms for 12-month (a), 24-month (b), and 36-month (c) OS prediction in the training set and for 12-month (d), 24-month (e), and 36-month (f) OS prediction in the validation set.

**Figure 8:**
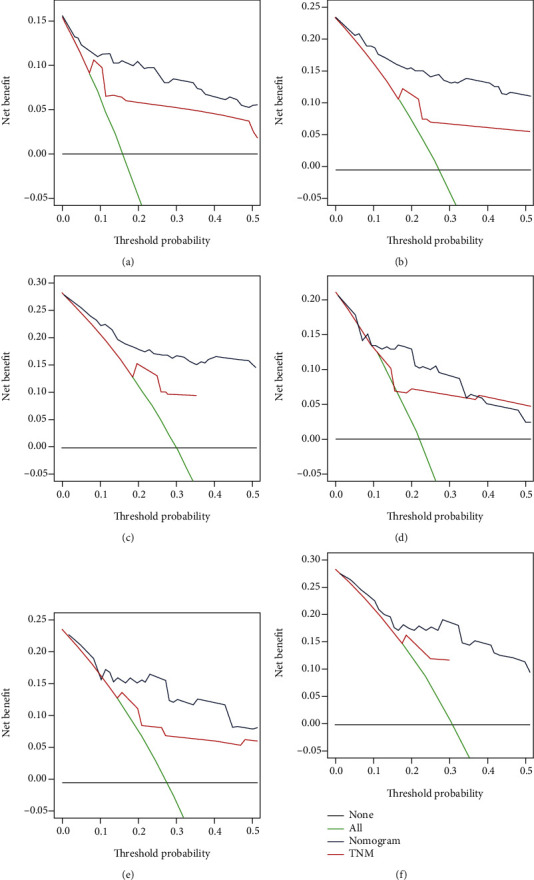
DCA of the nomograms for 12-month (a), 24-month (b), and 36-month (c) CSS prediction in the training set and for 12-month (d), 24-month (e), and 36-month (f) CSS prediction in the validation set.

The validation of these nomograms demonstrated that both two models performed well in predicting OS and CSS. In the training set, the AUCs of the nomogram predicting 12-, 24-, and 36-month OS were 0.849, 0.903, and 0.889, respectively (Figures 4(a)–4(c)). The AUCs of the nomogram predicting 12-, 24-, and 36-month CSS were 0.890, 0.880, and 0.881, respectively (Figures 5(a)–5(c)). In the validation set, the AUCs of the nomogram predicting 12-, 24-, and 36-month OS were 0.785, 0.829, and 0.818, respectively (Figures 4(d)–4(f)). The AUCs of the nomogram predicting 12-, 24-, and 36-month CSS were 0.847, 0.865, and 0.866, respectively (Figures 5(d)–5(f)). Additionally, the time-dependent ROC curves based on nomograms showed good performance in survival prediction and revealed that the proposed nomogram in the current study had a better predictive ability than TNM staging system in predicting OS and CSS at almost all time points (Figures 4(g) and 4(h), Figures 5(g) and 5(h)), and the AUCs of the single predictors of OS and CSS were significantly lower than those of the nomogram, suggesting that the prediction accuracy comprehensive model was better than separate clinicopathological feature. The calibration curves showed that predicted survival probability of 12-, 24-, and 36-month OS and CSS based on nomograms was almost consistent with actual observations in both sets (Figure 6). Besides, DCA was applied to compare the clinical usefulness between the novel prognostic model and traditional TNM staging system, and the results revealed that the established nomograms achieved greater net clinical benefits, meaning that it had better clinical implementation significance (Figure 7 and Figure 8).

### 4.4. Risk Classification Systems for OS and CSS

**Figure 9:**
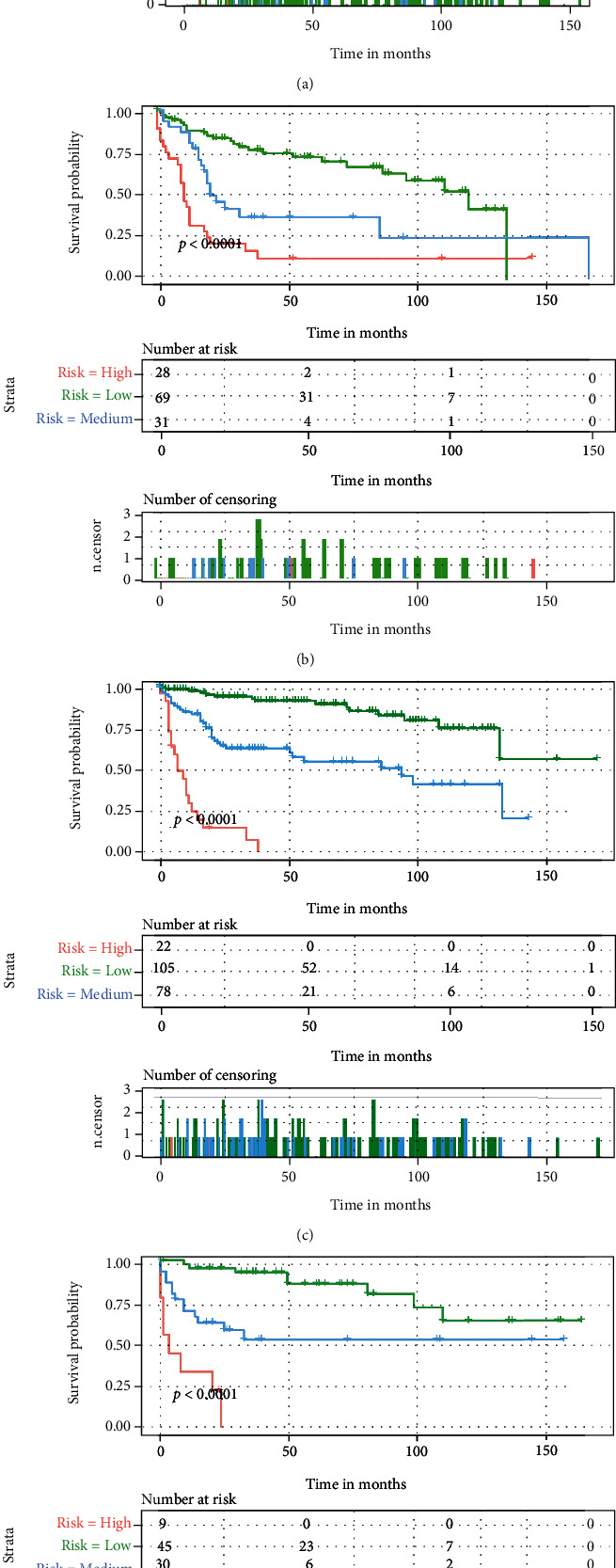
The Kaplan-Meier analysis of OS for patients stratified by the risk stratification system in the training set (a) and validation set (b) and of CSS for patients stratified by the risk stratification system in the training set (c) and validation set (d).

Furthermore, we calculated the risk scores based on the nomogram model for each elderly patient with POSNs to construct two risk classification systems and divided enrolled patients into three risk subgroups according to the cutoff analyses by the X-title program. The risk classification system of OS included low-risk group (score < 230), medium-risk group (score 230 ≤ nomogramscore ≤ 258), and high-risk group (score > 258), respectively. The risk classification system of CSS included low-risk group (score < 186), medium-risk group (score 186 ≤ nomogramscore ≤ 228), and high-risk group (score > 228), respectively. In the training group, the median OS time of the elderly patient with POSNs in the low-, medium-, and high-risk group was 99.0 months (95% CI, 78.0–120.0), 20.0 months (95% CI, 14.5–25.5), and 7.0 months (95% CI, 4.5–9.5), respectively. In the validation group, the median OS time of the elderly patient with POSNs in the low-, medium-, and high-risk groups was 106.0 months (95% CI, 77.0–135.0), 18.0 months (95% CI, 10.8–25.2), and 9.0 months (95% CI, 6.4–11.6), respectively. As shown in Figure 9, each risk subgroup represented a distinct prognosis and the OS and CSS in the three subgroups were accurately separated by these systems (all *P* < 0.001). Patients with high-risk scores had a worse prognosis than those with low-risk scores, indicating that the risk classification system constructed based on the nomogram has a significant predictive value for the prognosis of elderly patients with POSNs.

### 4.5. Development of a Web-Based Survival Rate Calculator

**Figure 10:**
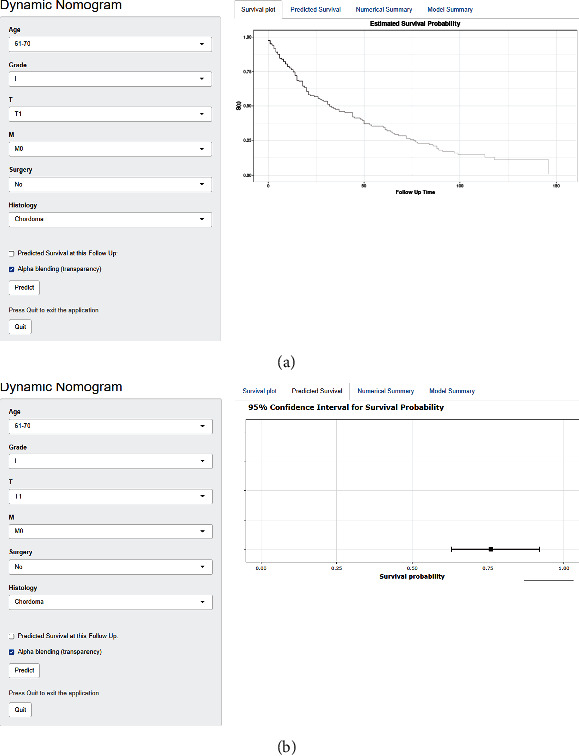
A web-based survival rate calculator. The graphical summary showed a rough range of survival rate (a). Numerical summary showed the survival probability at specific time point and its 95% confidence interval (b).

To contribute to personalized clinical decision-making, we further developed two web-based survival rate calculators based on proposed nomogram for calculating OS (https://research1.shinyapps.io/DynNomappOS/) and CSS (https://research1.shinyapps.io/DynNomappCSS/) of elderly patients with POSNs. The survival curve and the estimated survival probability were reported when users input the corresponding clinical features and specific time point on the left side of the web interface (Figure 10).

## 5. Discussion

As the world's population continues to age and the proportion of older persons in society is significantly increasing, the absolute number of elderly patients with POSNs is expected to increase [7]. While a previous study has made efforts towards evaluating the prognosis of patients with POSNs [11], the complexity and high incidence of elderly patients makes their targeted management and further research particularly important. Therefore, we developed two novel nomograms to provide personalized prediction for 12-, 24-, and 36-month OS and CSS of elderly patients with POSNs, which can act as a tool to select patients at high-risk of mortality and improve the management of this population. In this study, both models performed well in predicting survival probability, two nomograms demonstrated convinced predictive accuracy and great potential of clinical application. Besides, we found that these nomograms and nomogram-based risk classification systems were not inferior or even better than the current American Joint Committee on Cancer (AJCC) TNM classification system with regard to discriminatory power and were more quantitative and intuitive, which was more convenient and practical for clinicians to use. Noteworthy, DCA results indicated that the assessment of survival rate according to the nomogram led to more net benefit than based on TNM staging system. All in all, these results explicitly clarified the difference between the prognosis estimated using novel nomogram and that estimated by the conventional TNM staging system, which might explain the better ability of these nomograms in terms of survival prediction for elderly patients with POSNs than the TNM staging system. Furthermore, to facilitate the translation of these established models into clinical practice, we further developed two web-based survival rate calculators to allow better visualization and ease-of-use for clinicians. In contrast to the traditional nomogram that can only estimate survival probability for specific time periods, it can dynamically predict the probability of OS and CSS for elderly patients with POSNs at various time points [14].

In this study, age, T stage, M stage, grade, histological type, and surgery were determined to be independent prognostic factors for OS. Among these predictors, T stage, M stage, grade, histological type, and surgery were also found to be significantly correlated with CSS. Especially age itself, according to X-tile analysis, 70 years old and 80 years old were chosen as the optimal cutoff values, we believed the nomogram predicting OS of elderly patients with POSNs further opened up an opportunity to notice the significant role of age with much more subtle classification. The differential impact of age in overall survival versus cancer-specific survival led us to further reflect on the reasons for these differences. We found that OS of elderly patients with POSNs was significantly decreased with increasing chronological age, which fitted well with previous studies [11, 15]. While the age of onset of the disease did not seem to be significantly associated with CSS, in other words, the negative effect of advanced age was more apparent in OS compared to CSS among elderly patients with POSNs. This difference might be due to that the age-related organ dysfunction and decreased immunity made them tend to have various complications, and these debilitating diseases may reduce their chances of receiving radical surgery or other adjuvant therapies, some of them might even die directly from those additional diseases, thus reducing the probability of death attributed to POSNs [16, 17]. Besides, histological type was identified as an independent prognostic factor of OS and CSS, in a previous study, Zhou and colleagues reported the prognosis of patients diagnosed as primary spinal malignancies with different histological types which were varied [15]. A previous study demonstrated that overall median survival was histology-specific, where patients with osteosarcoma of the spine had the worst survival rate with the median OS of 18 months, which was in accordance with our findings [18]. In relation to tumor characteristics, the prognosis of elderly patients with POSNs was significantly correlated with the tumor extent, the condition of distant metastasis, and the degree of tumor differentiation. Higher T stage had been previously identified as a predictor of poor prognosis for several malignancies [19–21]. The T stage of the current AJCC TNM staging system for the case of malignant bone tumor occurring in the osseous spine is based on the extent of tumor invasion. In a population-based study of 1892 with primary osseous neoplasms of the spine, Mukherjee et al. investigated the influence of extent of local tumor invasion on survival outcome and confirmed that the perioperative identification of more extensive tumor invasion may portend a worse prognosis for patients with POSNs. [22]. Overexpression of matrix metalloproteinases made tumor more likely to invade into surrounding structures. In elderly population, the increased extent of tumor invasion may make them more difficult to remove surgically and may be more resistant to adjuvant therapies [23]. We also observed that the presence of metastatic disease was detrimental to the CSS and OS of elderly patients with POSNs, which was in line with a previous literature [11]. It was generally established that surgical intervention is the standard treatment strategy for POSNs. In a previous study investigating the association between surgical resection and survival rate in patients with POSNs, Mukherjee and colleagues suggested that patients undergoing surgical resection of primary spinal chordoma, chondrosarcoma, Ewing's sarcoma, and osteosarcoma exhibited prolonged survival independent of patient age, extent of local invasion, or location [18]. The improvement of survival in patients receiving surgery may be attributed to several factors, including tumor resection, local pain relief, spinal cord decompression, and spinal reconstruction [24–26]. In the present study, surgery was also determined to be an independent prognostic factor for OS and CSS, indicating its significant contribution in improving the survival outcome among elderly patients with POSNs.

However, some limitations should be acknowledged in the current study. Firstly, some potential factors and detailed treatment information were not integrated into our analysis due to the limited information available in the SEER database, such as lymphovascular invasion, genetic mutations, and chemotherapy regimens. Furthermore, since the rarity of POSNs, the external validation based on data from different populations of the models was not conducted, efforts are needed to collect prospective data to verify the predictive performance and general applicability of the nomograms.

## 6. Conclusion

This study developed and validated two nomograms and web-based survival rate calculators to estimate OS and CSS for elderly patients with POSNs. Compared with the 7th TNM staging, these novel nomograms were more accurate for survival prediction in this population, thus providing a novel reliable tool for risk assessment and assisting clinicians to make optimal care decisions.

## Figures and Tables

**Table 1:** The demographic and clinicopathologic information of elderly patients with POSNs in the OS group.

Variables	Total set (*N* = 430)		Training set (*N* = 302)		Validation set (*N* = 128)		*P* value
	*N*	%	*N*	%	*N*	%	
*Age*							
61-70	189	43.95	131	43.38	58	45.31	0.612
71-80	152	35.35	111	36.75	41	32.03	
>80	89	20.70	60	19.87	29	22.66	
*Race*							
Black	23	5.35	16	5.30	7	5.47	0.195
Other	32	7.44	18	5.96	14	10.94	
White	375	87.21	268	88.74	107	83.59	
*Sex*							
Female	183	42.56	129	42.72	54	42.19	1.000
Male	247	57.44	173	57.28	74	57.81	
*Histological type*							
Chordoma	184	42.79	127	42.05	57	44.53	0.622
Chondrosarcoma	138	32.09	101	33.44	37	28.91	
Osteosarcoma	50	11.63	32	10.60	18	14.06	
Other	58	13.49	42	13.91	16	12.50	
*Grade*							
Grade I	43	10.00	33	10.93	10	7.81	0.766
Grade II	67	15.58	48	15.89	19	14.84	
Grade III	49	11.40	36	11.92	13	10.16	
Grade IV	67	15.58	47	15.56	20	15.62	
Unknown	204	47.44	138	45.70	66	51.56	
*T stage*							
T1	227	52.79	167	55.30	60	46.88	0.236
T2	188	43.72	126	41.72	62	48.44	
T3	15	3.49	9	2.98	6	4.69	
*N stage*							
N0	415	96.51	289	95.70	126	98.44	0.259
N1	15	3.49	13	4.30	2	1.56	
*M stage*							
M0	383	89.07	264	87.42	119	92.97	0.129
M1	47	10.93	38	12.58	9	7.03	
*Marital status*							
Married	280	65.12	199	65.89	81	63.28	0.682
Unmarried	150	34.88	103	34.11	47	36.72	
*Tumor size(cm)*							
<6	141	32.79	100	33.11	41	32.03	0.976
6-12	206	47.91	144	47.68	62	48.44	
>12	83	19.30	58	19.21	25	19.53	
*Surgery*							
No	161	37.44	113	37.42	48	37.50	1.000
Yes	269	62.56	189	62.58	80	62.50	
*Radiotherapy*							
No	275	63.95	193	63.91	82	64.06	1.000
Yes	155	36.05	109	36.09	46	35.94	
*Chemotherapy*							
No	365	84.88	257	85.10	108	84.38	0.965
Yes	65	15.12	45	14.90	20	15.62	

**Table 2:** The demographic and clinicopathologic information of elderly patients with POSNs in the CSS group.

Variables	Total set (*N* = 289)		Training set (*N* = 205)		Validation set (*N* = 84)		*P* value
	*N*	%	*N*	%	*N*	%	
*Age*							
61-70	138	47.75	98	47.80	40	47.62	0.521
71-80	93	32.18	69	33.66	24	28.57	
>80	58	20.07	38	18.54	20	23.81	
*Race*							
Black	14	4.84	9	4.39	5	5.95	0.093
Other	23	7.96	12	5.85	11	13.10	
White	252	87.20	184	89.76	68	80.95	
*Sex*							
Female	118	40.83	83	40.49	35	41.67	0.853
Male	171	59.17	122	59.51	49	58.33	
*Histological type*							
Chordoma	141	48.79	102	49.76	39	46.42	0.925
Chondrosarcoma	96	33.22	68	33.17	28	33.33	
Osteosarcoma	18	6.23	12	5.85	6	7.14	
Other	34	11.76	23	11.22	11	13.10	
*Grade*							
Grade I	35	12.11	24	11.70	11	13.10	0.253
Grade II	44	15.22	26	12.68	18	21.43	
Grade III	28	9.69	19	9.27	9	10.71	
Grade IV	35	12.11	25	12.20	10	11.90	
Unknown	147	50.87	111	54.15	36	42.86	
*T stage*							
T1	161	55.71	110	53.66	51	60.71	0.523
T2	118	40.83	88	42.93	30	35.72	
T3	10	3.46	7	3.41	3	3.57	
*N stage*							
N0	279	96.54	198	96.59	81	96.43	0.947
N1	10	3.46	7	3.41	3	3.57	
*M stage*							
M0	259	89.62	185	90.24	74	88.10	0.587
M1	30	10.38	20	9.76	10	11.90	
*Marital status*							
Married	187	60.71	132	64.39	55	65.48	0.861
Unmarried	102	35.29	73	35.61	29	34.52	
*Tumor size(cm)*							
<6	101	34.95	66	32.20	35	41.67	0.250
6-12	135	46.71	98	47.80	37	44.05	
>12	53	18.34	41	20.00	12	14.28	
*Surgery*							
No	108	37.37	77	37.56	31	36.90	0.917
Yes	181	62.63	128	62.44	53	63.10	
*Radiotherapy*							
No	186	64.36	133	64.88	53	63.10	0.774
Yes	103	35.64	72	35.12	31	36.90	
*Chemotherapy*							
No	251	86.85	178	86.83	73	86.90	0.986
Yes	38	13.15	27	13.17	11	13.10	

**Table 3:** Univariate Cox analysis for OS and CSS of elderly patients with POSNs.

Variables	Univariate analysis for OS		Univariate analysis for CSS	
	HR (95% CI)	*P* value	HR (95% CI)	*P* value
*Age*				
61-70	Reference		Reference	
71-80	1.12 (0.79-1.6)	0.521	0.51 (0.27-0.93)	0.030
>80	2.49 (1.71-3.63)	<0.001	1.72 (0.96-3.06)	0.067
*Race*				
Black	Reference		Reference	
Other	0.67 (0.29-1.55)	0.347	1.74 (0.41-7.35)	0.453
White	0.63 (0.35-1.14)	0.13	1.17 (0.36-3.75)	0.796
*Sex*				
Female	Reference		Reference	
Male	1.11 (0.82-1.5)	0.504	1.25 (0.77-2.04)	0.369
*Histological type*				
Chordoma	Reference		Reference	
Chondrosarcoma	1.33 (0.91-1.95)	0.136	1.68 (0.94-3.02)	0.081
Osteosarcoma	6.14 (3.81-9.89)	<0.001	4.56 (2.08-9.99)	<0.001
Other	4.25 (2.77-6.51)	<0.001	5.11 (2.65-9.85)	<0.001
*Grade*				
I	Reference		Reference	
II	1.73 (0.84-3.55)	0.138	7.41 (0.93-59.26)	0.059
III	4.4 (2.18-8.88)	<0.001	10.7 (1.31-87.17)	0.027
IV	7.07 (3.6-13.9)	<0.001	26.58 (3.54-199.59)	0.001
Unknown	2.11 (1.11-3.99)	0.022	8.56 (1.17-62.52)	0.034
**T stage**				
T1	Reference		Reference	
T2	1.48 (1.09-2.02)	0.013	1.83 (1.12-3)	0.017
T3	3.9 (1.88-8.07)	<0.001	6.48 (2.46-17.04)	<0.001
*N stage*				
N0	Reference		Reference	
N1	2.21 (1.2-4.07)	0.011	1.9 (0.69-5.23)	0.216
*M stage*				
M0	Reference		Reference	
M1	6.33 (4.24-9.46)	<0.001	9.29 (5.15-16.76)	<0.001
*Marital status*				
Married	Reference		Reference	
Unmarried	1.08 (0.79-1.47)	0.637	0.77 (0.46-1.28)	0.312
*Tumor size* (*cm*)				
<6	Reference		Reference	
6-12	1.16 (0.82-1.63)	0.395	1 (0.57-1.76)	0.999
>12	1.35 (0.88-2.07)	0.165	1.79 (0.94-3.4)	0.075
*Surgery*				
No	Reference		Reference	
Yes	0.38 (0.28-0.51)	<0.001	0.28 (0.17-0.45)	<0.001
*Radiotherapy*				
No	Reference		Reference	
Yes	1.49 (1.1-2.03)	0.01	1.49 (0.92-2.41)	0.108
*Chemotherapy*				
No	Reference		Reference	
Yes	2.22 (1.52-3.24)	<0.001	3.39 (2-5.73)	<0.001

## Data Availability

The datasets generated during and/or analyzed during the current study are available from the corresponding author on reasonable request.

## Abbreviations

POSNs:Primary osseous spinal neoplasms

SEER:Surveillance, Epidemiology, and End Results

ROC:Receiver operating characteristic curve

AUC:Area under the curve

DCA:Decision curve analyses

OS:Overall survival

CSS:Cancer-specific survival

AJCC:American Joint Committee on Cancer

CI:Confidence interval

HR:Hazard ratio.

## References

[1] Fisher C., Keynan O., Boyd M. C., Dvorak M. F. (2005). The surgical management of primary tumorsof the spine: initial results of an ongoing prospective cohort study. *Spine*.

[2] Meng T., Yin H., Li B. (2015). Clinical features and prognostic factors of patients with chordoma in the spine: a retrospective analysis of 153 patients in a single center. *Neuro-Oncology*.

[3] Yin H., Cheng M., Li B. (2015). Treatment and outcome of malignant giant cell tumor in the spine. *Journal of Neuro-Oncology*.

[4] Yin H., Zhou W., Meng J. (2014). Prognostic factors of patients with spinal chondrosarcoma: a retrospective analysis of 98 consecutive patients in a single center. *Annals of Surgical Oncology*.

[5] Donthineni R. (2009). Diagnosis and staging of spine tumors. *Orthopedic Clinics of North America*.

[6] Sundaresan N., Boriani S., Rothman A., Holtzman R. (2004). Tumors of the osseous spine. *Journal of Neuro-Oncology*.

[7] WHO (2018). Ageing and Life Course. http://www.who.int/ageing/en/.

[8] Campisi J. (2013). Aging, cellular senescence, and cancer. *Annual Review of Physiology*.

[9] Triana-Martínez F., Pedraza-Vázquez G., Maciel-Barón L. A., Königsberg M. (2016). Reflections on the role of senescence during development and aging. *Archives of Biochemistry and Biophysics*.

[10] Loaiza N., Demaria M. (2016). Cellular senescence and tumor promotion: Is aging the key. *Biochimica et biophysica acta. Reviews on cancer*.

[11] Zhou Q., Li A., Lin Z., Zhang H. J. S. (2020). A nomogram and a risk classification system predicting the cancer-specific survival of patients with initially-diagnosed osseous spinal and pelvic tumors. *Spine*.

[12] Helenius I. J., Krieg A. H. (2021). Primary malignant bone tumours of spine and pelvis in children. *Journal of Children's Orthopaedics*.

[13] Camp R., Dolled-Filhart M., Rimm D. L. (2004). X-tile: a new bio-informatics tool for biomarker assessment and outcome-based cut-point optimization. *Clinical Cancer Research*.

[14] Iasonos A., Schrag D., Raj G. V., Panageas K. S. (2008). How To Build and Interpret a Nomogram for Cancer Prognosis. *Journal of Clinical Oncology*.

[15] Zhou L., Huang R., Wei Z., Meng T., Yin H. (2021). The Clinical Characteristics and Prediction Nomograms for Primary Spine Malignancies. *Frontiers in Oncology*.

[16] DeSantis C., Miller K., Dale W. (2019). Cancer statistics for adults aged 85 years and older, 2019. *CA: A Cancer Journal for Clinicians*.

[17] Skaznik-Wikiel M., Sukumvanich P., Austin R. (2012). Heavy cervical cancer burden in elderly women: how can we improve the situation. *Acta Cytologica*.

[18] Mukherjee D., Chaichana K. L., Parker S. L., Gokaslan Z. L., McGirt M. J. (2013). Association of surgical resection and survival in patients with malignant primary osseous spinal neoplasms from the Surveillance, Epidemiology, and End Results (SEER) database.

[19] Shao C. Y., Yu Y., Li Q. F. (2021). Development and Validation of a Clinical Prognostic Nomogram for Esophageal Adenocarcinoma Patients. *Frontiers in Oncology*.

[20] Jiang A., Liu N., Zhao R. (2021). Construction and validation of a novel nomogram to predict the overall survival of patients with combined small cell lung cancer: A Surveillance, Epidemiology, and End Results Population-Based Study. *Cancer Control*.

[21] Guo Q., Wang Y., An J., Wang S., Dong X., Zhao H. (2021). A Prognostic Model for Patients With Gastric Signet Ring Cell Carcinoma. *Technology in Cancer Research & Treatment*.

[22] Mukherjee D., Chaichana K. L., Adogwa O. (2011). Association of extent of local tumor invasion and survival in patients with malignant primary osseous spinal neoplasms from the surveillance, epidemiology, and end results (SEER) database. *World Neurosurgery*.

[23] Gokaslan Z. L., Chintala S. K., York J. E. (1998). Expression and role of matrix metalloproteinases MMP-2 and MMP-9 in human spinal column tumors. *Clinical & Experimental Metastasis*.

[24] Fisher C. G., Saravanja D. D., Dvorak M. F. (2011). Surgical management of primary bone tumors of the Spine. *Spine*.

[25] Orguc S., Arkun R. (2014). Primary tumors of the spine. *Seminars in Musculoskeletal Radiology*.

[26] Ozturk A., Gokaslan Z. L., Wolinsky J. P. (2014). Surgical treatment of sarcomas of the spine. *Current Treatment Options in Oncology*.

